# Cancer Care Delivery and Financial Barriers Among Patients in Lebanon: A Descriptive Multicenter Cross-Sectional Study

**DOI:** 10.7759/cureus.115137

**Published:** 2026-08-25

**Authors:** Ghadir M Nasreddine, Nadeen Zayour, Jacqueline Najjar, Elie Daibess, Elie j Karam, Zeinab Hammoud, Solay Farhat, Mohamad Ali Hachem, Mohamad Abbass, Zeinab Sleiman, Ahmad Ibrahim, Issam Chehade, Maroun Sadek, Bassam Matar

**Affiliations:** 1 Department of Internal Medicine, Division of Hematology and Oncology, Faculty of Medical Sciences, Lebanese University, Beirut, LBN; 2 Organized Research Unit, Al Zahraa Hospital University Medical Center, Beirut, LBN; 3 International Department, Gustave Roussy, Villejuif, FRA; 4 Department of Internal Medicine, Faculty of Medical Sciences, Lebanese University, Beirut, LBN; 5 Department of Hematology and Oncology, Makassed General Hospital, Beirut, LBN; 6 Department of Hematology and Oncology, Rafik Hariri University Hospital, Beirut, LBN; 7 Department of Hematology and Oncology, Lebanese Hospital Geitaoui-University Medical Center, Beirut, LBN; 8 Department of Hematology and Oncology, Al Zahraa Hospital University Medical Center, Beirut, LBN

**Keywords:** access to care, cancer care, guideline-concordant care, health coverage, lebanon, socioeconomic status, treatment delay

## Abstract

Background

Cancer care in Lebanon is provided within a fragmented health system affected by economic instability, incomplete insurance coverage, and variable access to diagnostic and treatment services. These factors may influence the timely initiation of treatment, continuity of care, and the delivery of evidence-based oncology care. This study aimed to characterize access to cancer care in Lebanon by evaluating treatment delays, socioeconomic burdens, health coverage, treatment supply sources, and concordance with National Comprehensive Cancer Network (NCCN) recommendations among adults receiving hospital-based cancer therapy.

Methods

A descriptive cross-sectional multicenter study was conducted in five hospitals in Lebanon between July 2024 and June 2025. A total of 244 adult Lebanese patients receiving active cancer treatment were included. Data were collected using a structured questionnaire that covered socioeconomic characteristics, cancer-related factors, and treatment-related factors.

Results

Most participants were women (n=144, 59.0%), and the largest age group was 60-74 years (n=113, 46.3%). More than half were unemployed (n=138, 56.6%) and lacked medical insurance coverage (n=139, 57.0%). Household income substantially decreased for 80.7% (n=197) of the participants, with reliance on multiple coping strategies, including family support, savings, debt, donations, and asset sales. Breast cancer was the most common diagnosis (n=66, 27.1%), followed by colorectal (n=37, 15.2%) and lung (n=36, 14.8%) cancer. Advanced-stage disease was common, with 50.4% (n=123) presenting with stage IV disease and 22.1% (n=54) presenting with stage III disease. Treatment initiation delays exceeding two weeks were reported by 52.5% (n=128) of the participants, mainly related to pre-treatment procedures, financial constraints, and diagnostic delays. Most patients received their planned cancer therapies, with the treatment supplied primarily by hospitals and the Ministry of Public Health. Overall, 84.8% (n=207) of patients received NCCN guideline-concordant treatment.

Conclusions

Hospital-based cancer care in Lebanon remains limited by socioeconomic barriers and incomplete financial protection. However, among patients who accessed active oncology care, guideline-concordant treatment was largely maintained. These findings underscore important challenges in financial protection, diagnostic pathways, and continuity of oncology services that warrant further attention in Lebanon.

## Introduction

Cancer remains one of the leading causes of morbidity and mortality worldwide and represents an increasing public health challenge. In 2022, an estimated 20 million new cancer cases and 9.7 million cancer-related deaths occurred worldwide [1]. Population growth, population aging, and increased exposure to cancer risk factors are expected to further increase the global cancer burden. Moreover, advances in prevention, early detection, treatment, and supportive care have improved cancer outcomes. However, these benefits are not equitably distributed within and between countries [1]. Populations living in limited-resource healthcare systems often experience later-stage diagnosis, a shortage of cancer medicines, and greater dependency on out-of-pocket payments for medical care [2]. Timely initiation of treatment after diagnosis is an important component of high-quality cancer care. Nevertheless, delays may occur prior to the cancer treatment pathway, including specialist consultation, imaging, pathology reporting, immunohistochemistry and molecular testing, multidisciplinary assessment, treatment approval, and treatment acquisition [3]. Timely initiation of cancer treatment is a recognized quality indicator in oncology, as delays at different points along the care pathway may adversely affect patient outcomes [3].

Cancer treatment requires multiple interconnected highly demanding steps, including pathological diagnosis, staging investigations, molecular testing, possible surgical intervention, laboratory monitoring and repeated hospital admission for systemic treatment. The development of newer cancer therapies, such as targeted therapies, immune checkpoint inhibitors, and antibody-drug conjugates, has improved outcomes but has also contributed to increasing treatment costs for patients and families [4,5]. The burden of cancer extends beyond clinical complications and treatment toxicities and may result in financial toxicity due to direct and indirect costs [6]. Consequently, households may experience increased expenditures and rely on support from family members or community organizations to continue treatment. Such coping strategies may have long-term socioeconomic consequences and may contribute to treatment disruption or discontinuation [2].

Cancer treatment recommendations are guided by tumor type, disease stage, pathology, molecular characteristics, performance status, comorbidities, previous treatments, and evidence-based clinical practice guidelines such as those developed by the National Comprehensive Cancer Network (NCCN) [7]. However, deviations from guideline recommendations do not always indicate poor-quality care, as treatment modifications may be clinically appropriate due to contraindications, comorbidities, toxicity, or patient preferences. Conversely, non-concordance may also result from health-system barriers, including medication unavailability, lack of coverage, delayed authorization, inability to perform molecular testing, or financial constraints [8,9].

In Lebanon, the cancer burden is considerable. According to the Global Cancer Observatory, in 2022, Lebanon had 13,034 new cancer cases and 7307 cancer deaths, reflecting the need for a broad range of systemic treatment services [10]. The financial and socioeconomic situation in Lebanon since the onset of the economic crisis in 2019 has increased challenges to the health care system [11]. Lebanon has had to contend with the crisis’s impact on the availability and prices of essential therapies, since the country relies on imported pharmaceuticals and medical supplies [12]. In Lebanon, cancer care is delivered in a pluralistic health system in which public and private providers and different financing institutions coexist. Patients may pay directly out-of-pocket or may be covered by the Ministry of Public Health (MOPH), the National Social Security Fund (NSSF), private insurance companies, security institutions, or non-governmental organizations (NGOs). However, approval processes and drug coverage differ across these financing systems [5]. Fragmentation of the healthcare system can create a gap between what the physician recommends and what the patient receives. In some cases, patients must pay the costs upfront and seek reimbursement afterward, but the reimbursement may cover only part of the costs. As a result, some patients may rely on multiple sources of financing to obtain cancer treatment [5,13].

Comprehensive data describing the clinical, socioeconomic, and treatment-related aspects of cancer care are essential to characterize current challenges and inform future cancer control strategies in Lebanon. However, multicenter data regarding these aspects of cancer care and concordance with evidence-based treatment recommendations remain limited in Lebanon [13,14]. Therefore, the primary objective of the current study was to characterize the cancer care landscape among Lebanese adults receiving hospital-based cancer treatment. The secondary objectives were to describe patients’ clinical and socioeconomic characteristics, assess treatment delays and treatment modalities, examine sources of treatment coverage and treatment supply, and evaluate concordance with NCCN guideline recommendations.

## Materials and methods

Study design

This descriptive, multicenter, cross-sectional survey was conducted among adult cancer patients receiving active treatment at five hospitals in Lebanon: Al Zahraa Hospital University Medical Center, Saint George Hospital University Medical Center, Rafik Hariri University Hospital, Makassed General Hospital, and Lebanese Geitaoui Hospital. These hospitals were selected because they provide hospital-based oncology services and serve patients with diverse health coverage from various Lebanese governorates. Data collection was carried out between July 13, 2024, and June 27, 2025.

Study population and data collection procedure

Eligible participants were Lebanese adults aged ≥18 years of either sex with a confirmed diagnosis of cancer who were receiving active anticancer treatment at one of the five participating hospitals. Patients were excluded if they were pregnant, declined to participate, or were receiving only blood transfusions for hematological conditions without active anticancer therapy. Patients who met the eligibility criteria were invited to participate by an oncology fellow during their hospital visit. Of the 412 eligible patients approached, 244 agreed to participate and 168 declined participation. After providing informed consent, the participants completed a structured questionnaire. The structured questionnaire was developed by the study team based on the study objectives and relevant literature. It was reviewed by members of the research team with expertise in oncology and clinical research to ensure content relevance and clarity and was pilot-tested on 15 patients before initiation of formal data collection. Feedback from the pilot testing was used to refine the wording and organization of the questionnaire. The final questionnaire included sections covering sociodemographic characteristics, clinical and cancer-related variables (including cancer type and stage), treatment characteristics, and other variables relevant to the study objectives.

Study variables

The study variables were grouped into five domains: sociodemographic, socioeconomic, clinical, treatment-related, and cost-related. The socioeconomic variables included monthly household income, the perceived impact of the ongoing Lebanese economic crisis on household income, sources of income, financial coping strategies, health insurance status, and type of medical coverage. The clinical variables included cancer type, disease stage, and time since cancer diagnosis. The treatment-related variables included treatment modality, treatment supply, and treatment delay. Treatment delay was defined as the initiation of hospital-administered systemic anticancer therapy more than two weeks after cancer diagnosis. A literature review conducted by the study team found no universally accepted definition or standardized cutoff for treatment delay. Therefore, the two-week threshold was selected a priori as an operational definition based on the available literature and expert consensus within the study team. The duration of the delay and reported reasons for delay were recorded. The cost-related variables included out-of-pocket expenditures for genetic testing, surgery, laboratory investigations, and the treatment payment method. Concordance with the NCCN recommendations was assessed by comparing the treatment received with the corresponding disease-specific NCCN guidelines applicable during the study period [7]. The assessment considered the cancer type, stage, and documented clinical characteristics relevant to treatment selection.

Treatment was classified as NCCN-concordant when the documented treatment was consistent with the recommended management options in the applicable NCCN guideline. Clinically appropriate deviations were considered concordant when the documented treatment represented an alternative regimen or management strategy explicitly recognized as an appropriate option within the applicable NCCN guideline. Cases that could not be matched to a recommended treatment option were classified as non-NCCN-concordant. The guideline version used for each cancer type was recorded according to the version applicable during the patient's treatment period.

Bias control strategies

Several potential sources of bias were considered. Selection bias may have occurred because the study included only patients receiving active anticancer treatment at the participating hospitals. Consequently, patients who were unable to access treatment or were treated exclusively with oral anticancer therapies outside the hospital setting may have been underrepresented. Recall bias was possible because financial information and reasons for treatment delay were self-reported. To minimize information bias, data were collected using a standardized, structured questionnaire administered by trained oncology fellows. All data collectors received standardized training before study initiation, and clinical information was verified against patients' medical records whenever available to ensure data accuracy.

Sampling approach and sample size

A convenience sampling approach was used. All eligible patients attending the participating hospitals during the study period were invited to participate. Recruitment continued throughout the study period, and a total of 244 patients were enrolled.

Ethical approval and considerations

The study was conducted in accordance with the principles of the Declaration of Helsinki. Ethical approval was obtained from the Institutional Review Board (IRB) of each participating hospital before study initiation: Al Zahraa Hospital University Medical Center (approval no. 22/2023), Saint George Hospital University Medical Center (approval no. IRB-REC/O/013-25/0925), Rafik Hariri University Hospital (approval no. 2024-0205), Makassed General Hospital (approval no. 230124) and Lebanese Geitaoui Hospital (approval no. 2024-IRB-03). Written informed consent was obtained from all participants or their legal representatives prior to enrollment. Participation was voluntary, patients were informed that they could withdraw from the study at any time without affecting their medical care and that the confidentiality of their personal information would be maintained.

Statistical analysis

All study variables were categorical and are presented as frequencies and percentages. Statistical analyses were performed using IBM SPSS Statistics for Windows, Version 29 (Released 2022; IBM Corp., Armonk, New York, United States).

## Results

Sociodemographic and socioeconomic characteristics

A total of 244 participants were included in the analysis. The majority were female patients (59.0%, n=144), whereas 41.0% (n=100) were male patients. The participants were predominantly older adults, nearly half of whom were aged 60-74 years (46.3%, n=113), followed by those aged 45-59 years (26.2%, n=64) and 75-90 years (13.9%, n=34). Younger age groups were less represented, including 30-44 years (9.8%, n=24) and 18-29 years (3.7%, n=9). Regarding educational attainment, 41.4% (n=101) reported having higher education, 21.3% (n=52) had completed secondary education, and 18.9% (n=46) had a high school education. Lower education levels were less common, with 13.5% (n=33) having primary education and 4.9% (n=12) being illiterate. In terms of employment status, more than half of the participants were unemployed (56.6%, n=138). Nearly 30% (29.5%, n=72) were employed, 11.9% (n=29) were retired, and 2.1% (n=5) were students. Geographically, 30.3% (n=74) resided in Beirut, whereas the majority lived in other governorates (69.7%, n=170). Most participants were currently married (74.2%, n=181).

The monthly household income distribution is also presented in Table 1.

**Table 1:** Sociodemographic and socioeconomic characteristics of the study participants (n=244) ^¥^Percentages do not sum to 100%, as participants could report multiple income sources; ^a^Monthly salary referred to the participant's own salary and/or the salary of one or more household member; ^b^Selling assets included the sale of personal belongings, gold, land, or other valuable property.

Variables	n	%
Gender		
Female	144	59.02
Male	100	40.98
Age group (years)		
18-29	9	3.69
30-44	24	9.84
45-59	64	26.23
60-74	113	46.31
75-90	34	13.93
Educational attainment		
Illiterate	12	4.92
Primary education	33	13.52
Secondary education	52	21.31
High school education	46	18.85
Higher education	101	41.39
Occupational status		
Employed	72	29.51
Retired	29	11.89
Student	5	2.05
Unemployed	138	56.56
Governorate		
Beirut	74	30.33
Other governorates	170	69.67
Marital status		
Currently married	181	74.18
Other (divorced, single, widowed)	63	25.82
Household income		
<200$	10	4.1
200-500$	78	31.97
500-700$	53	21.72
700-1000$	36	14.75
1000-1500$	32	13.11
>1500$	35	14.34
Effect of economic crisis in Lebanon on household income		
Household income unaffected	29	11.89
Household income decreased a bit	18	7.37
Household income decreased harshly	197	80.74
Household income source^¥^		
Monthly salary^a^	113	46.31
Assistance from relatives	74	30.33
Daily work	41	16.8
Selling assets^b^	35	14.34
Savings	31	12.7
Retirement income	29	11.89
Transfers	29	11.89
Debts	27	11.07
Donations	21	8.61
Landlord	6	2.46
Loan	5	2.05
Owns a store	18	7.38
Medical insurance		
No	139	56.97
Yes	105	43.03
Medical insurance coverage (n=105)		
Hospital fees only	22	20.95
Treatment only	4	3.8
Hospital fees and treatment	72	68.57
None	7	6.67

Nearly 32.0% (n=78) reported a monthly income between USD 200-500, followed by 21.7% (n=53) earning USD 500-700. Approximately 14.8% (n=36) reported incomes between USD 700-1000, and 13.1% (n=32) earned USD 1000-1500. A smaller proportion reported incomes above USD 1500 (14.3%, n=35), whereas 4.1% (n=10) reported earning less than USD 200 per month. Most participants reported a decrease in household income following the ongoing Lebanese economic crisis.

More than four-fifths of the participants (80.7%, n=197) reported that their household income had decreased harshly. Only 11.9% (n=29) reported no change in income, whereas 7.3% (n=18) experienced a slight decrease. However, the most common household income source was a monthly salary (46.3%, n=113), followed by assistance from relatives (30.3%, n=74). Additional coping strategies included daily work (16.8%, n=41), selling assets (14.3%, n=35), and using savings (12.7%, n=31). Other reported sources included retirement income (11.9%, n=29), transfers (11.9%, n=29), and debt accumulation (11.1%, n=27). Smaller proportions reported reliance on donations (8.6%, n=21), owning a store (7.4%, n=18), landlord income (2.5%, n=6), or loans (2.1%, n=5).

With respect to health coverage, more than half of the participants were uninsured (57.0%, n=139). Among those reporting medical insurance (43.0%, n=105), coverage varied: 68.6% (n=72) had coverage for both hospital fees and treatment, 21% (n=22) had hospital fees only, and 3.8% (n=4) had treatment-only coverage. A small proportion (6.7%, n=7) reported having insurance but with no effective coverage (Table 1).

Cancer-related clinical characteristics

Types of Cancer

Breast cancer was the most frequently reported diagnosis, accounting for 27.1% (n=66) of the cases. This was followed by colorectal cancer (15.2%, n=37) and lung cancer (14.8%, n=36). Ovarian cancer represented 7.4% (n=18) of the cases, whereas non-Hodgkin lymphoma accounted for 4.5% (n=11). A substantial proportion of participants (31.2%, n=76) reported other types of cancer (Table 2).

**Table 2:** Clinical characteristics and treatment initiation barriers ^¥^Percentages do not sum to 100%, as participants could report multiple reasons for delays in treatment initiation.

Variables	n	%
Type of cancer		
Breast cancer	66	27.05
Colorectal cancer	37	15.16
Lung cancer	36	14.75
Ovarian cancer	18	7.38
Non-Hodgkin Lymphoma	11	4.51
Other types	76	31.16
Stage of cancer		
Not staged	13	5.33
Stage I	15	6.15
Stage II	39	15.98
Stage III	54	22.13
Stage IV	123	50.41
Time since cancer diagnosis		
Less than 3 months	101	41.39
3–6 months	45	18.44
6–12 months	41	16.8
1–2 years	28	11.48
2–5 years	23	9.43
More than 5 years	6	2.46
Delay in treatment initiation		
No delay	116	47.54
Yes	128	52.46
Duration of delay (n=128)		
2-4 weeks	52	40.62
4-6 weeks	25	19.53
6-8 weeks	15	11.71
8-10 weeks	9	7.03
10 to 12 weeks	7	5.46
>12 weeks	20	15.62
Reasons for delay^¥^ (n=128)		
Pre-treatment surgery	59	46.09
Financial constraints	51	39.84
Delay in imaging reports	36	28.12
Delay in lab tests	33	25.78

Stage of Cancer

More than half of the participants were diagnosed at an advanced stage, with stage IV cancer being the most common stage (50.4%) of the sample, followed by stage III (22.1%) and stage II (16.0%). Early-stage diagnoses were less common, with stage I reported by 6.2% (n=15). A small proportion (5.3%, n=13) reported that their cancer stage was not determined at the time of the survey (Table 2).

Time Since Cancer Diagnosis

Most participants reported a recent cancer diagnosis, with 41.4% (n=101) diagnosed within the past three months. An additional 18.4% (n=45) had been diagnosed between three to six months, and 16.8% (n=41) had been diagnosed between six and 12 months. A longer duration since diagnosis was less common, with 11.5% (n=28) diagnosed one to two years prior, 9.4% (n=23) diagnosed two to five years earlier, and only 2.5% (n=6) diagnosed more than five years ago (Table 2).

Treatment Initiation Delays

More than half of the participants (52.5%, n=128) reported experiencing a delay in initiating cancer treatment. Among those reporting delays, the most common delay duration was two to four weeks (40.6%, n=52), followed by four to six weeks (19.5%, n=25) and six to eight weeks (11.7%, n=15). Longer delays were also reported, including eight to 10 weeks (7.0%, n=9), 10-12 weeks (5.5%, n=7), and more than 12 weeks (15.6%, n=20). Among participants who experienced delays, the most frequently cited reason was pre-treatment surgery (46.1%, n=59). Financial constraints were reported by 39.8% (n=51) of the participants as contributing factors to treatment initiation delays. Diagnostic-related delays, including delays in imaging reports (28.1%, n=36) and laboratory testing delays (25.8%, n=33), were also reported (Table 2).

Medical costs and receipt of treatment

The indirect and direct medical costs experienced by the participants are summarized in Table 3.

**Table 3:** Stratified direct and indirect medical costs and receipt of treatment NSSF: National Social Security Fund; MOPH: Ministry of Public Health. ^§^Participants could receive a single treatment modality or a combination of treatment modalities; therefore, percentages do not sum to 100%; ^¥^Percentages do not sum to 100%, as participants could report multiple payment methods.

Parameter	n	%
Indirect medical cost		
Genetic testing performed		
No	169	69.26
Yes	75	30.74
Underwent surgery before treatment		
No	147	60.25
Yes	97	39.75
Approximate amount paid for surgery (n=97)		
1,000 - 3,000$	28	28.86
3,000 - 5,000$	7	7.21
5,000 - 10,000$	11	11.34
10,000 - 20,000$	9	9.27
20,000 - 30,000$	2	2.06
Covered by insurance and/or the NSSF, etc.	40	41.23
Direct Medical Cost		
Cost of laboratory tests		
10-40$	49	20.08
41-70$	34	13.93
71 -110$	87	35.66
111 -150$	4	1.64
>150$	70	28.69
Treatment Modality^§ ^and receipt		
Chemotherapy		
Fully received	187	76.64
Not received or reduced dose	14	5.74
Undergoing another therapy	43	17.62
Targeted therapy		
Fully received	71	29.1
Not received or reduced dose	5	2.05
Undergoing another therapy	168	68.85
Immunotherapy		
Fully received	38	15.57
Not received or reduced dose	5	2.05
Undergoing another therapy	201	82.38
Radiotherapy		
Fully received	9	3.69
Undergoing another therapy	235	96.31
Payment Method for Treatment^¥^		
Cash	159	65.16
Medical insurance	112	45.9
Donations	81	33.19
NSSF	36	14.75
MOPH	36	14.75

Genetic testing was performed in 30.7% (n=75) of the participants, whereas the majority (69.3%, n=169) reported not undergoing genetic testing. Prior to initiating treatment, 39.8% (n=97) of the participants underwent surgery, whereas 60.3% (n=147) did not. Among participants who reported surgical intervention, out-of-pocket expenditures varied considerably. Payments of USD 1,000-3,000 were most common (28.9%, n=28), followed by USD 5,000-10,000 (11.3%, n=11) and USD 10,000-20,000 (9.2%, n=9). Smaller proportions reported expenditures of USD 3,000-5,000 (7.2%, n=7) or USD 20,000-30,000 (2.1%, n=2). Notably, 41.2% (n=40) reported that surgical costs were fully covered by medical insurance and/or the National Social Security Fund (NSSF).

The reported costs of diagnostic laboratory testing varied among participants. The most frequently reported cost category was USD 71-110, reported by 35.7% (n=87) of participants, whereas 28.7% (n=70) reported laboratory costs exceeding USD 150.

With respect to treatment modalities, four modalities were assessed. Among the participants for whom chemotherapy was indicated, 76.6% (n=187) received chemotherapy as planned, whereas 5.7% (n=14) did not receive chemotherapy or received a reduced dose, and 17.6% (n=43) received an alternative therapeutic modality. Among the participants for whom targeted therapy was indicated, 29.1% (n=71) received the treatment as planned, whereas 2.1% (n=5) did not receive treatment or received a reduced dose. Similarly, among those for whom immunotherapy was indicated, 15.6% (n=38) received treatment as planned, whereas 2.1% (n=5) did not receive immunotherapy. Radiotherapy was received as planned by 3.7% (n=9) of the participants for whom it was indicated.

Multiple sources of payment for cancer treatment were reported among participants. Out-of-pocket cash payments were the most frequently used source, reported by 65.2% (n=159) of the participants, followed by medical insurance (45.9%, n=112) and donations (33.2%, n=81). Public and institutional coverage sources included the NSSF (14.8%, n=36) and the Ministry of Public Health (MOPH; 14.8%, n=36) (Table 3).

Treatment availability and sources of supply

Table 4 describes the availability of cancer therapies and the sources of supply by therapy type.

**Table 4:** Treatment supply status and sources of drug supply ^a^Direct out-of-pocket expense. MOPH: Ministry of Public Health

Treatment	Chemotherapy (n=201)	Targeted therapy (n=76)	Immunotherapy (n=43)	Radiotherapy (n=9)
Status				
Not supplied	1 (0.5%)	3 (3.9%)	5 (11.6%)	0 (0.0%)
Supplied	200 (99.5%)	73 (96.1%)	38 (88.4%)	9 (100%)
Source of supplied drug				
Hospital supply	143 (71.5%)	43 (58.9%)	31 (81.6%)	-
MOPH supply	21 (10.5%)	18 (24.7%)	4 (10.5%)	
Pharmacy purchase^a^	20 (10.0%)	6 (8.2%)	2 (5.3%)	
Community supply	2 (1.0%)	1 (1.4%)	1 (2.6%)	
Guarantor insurance	0 (0.0%)	1 (1.4%)	0 (0.0%)	
Army supply	2 (1.0%)	2 (2.7%)	0 (0.0%)	
Multiple sources	12 (6.0%)	2 (2.7%)	0 (0.0%)	

Overall, the vast majority of patients received their prescribed treatments, with supply rates of 99.5% for chemotherapy, 96.1% for targeted therapy, 88.4% for immunotherapy, and 100% for radiotherapy treatment. Among patients who were supplied with their respective drug therapy, hospitals were the predominant source across all therapy modalities, accounting for 71.5% of the chemotherapy supply, 58.9% of the targeted therapy supply, and 81.6% of the immunotherapy supply. The MOPH supply represented the second most common source, particularly for targeted therapy (24.7%), whereas out-of-pocket pharmacy purchases accounted for 10.0% of the chemotherapy and 8.2% of the targeted therapy supplies. Community donation supply, health insurance, and military sources contributed marginally across all therapy types (Table 4).

Concordance with NCCN recommendations

Among the 244 patients included in the analysis, 207 (84.8%) received treatment concordant with the applicable NCCN recommendations, whereas 37 (15.2%) received treatment that was classified as non-NCCN concordant.

## Discussion

This multicenter descriptive cross-sectional study examines the clinical, economic, and treatment-access aspects among adult cancer patients receiving active therapy across five hospitals in Lebanon. The study population consisted predominantly of older adults, with patients aged 60-74 years representing the largest age group. This observation is consistent with the well-established epidemiology of cancer, in which the incidence of malignancy increases with advancing age owing to the cumulative effects of genetic alterations, environmental exposures, and biological aging processes [4,15].

The clinical profile of the study population revealed that breast cancer was the most frequently reported malignancy, followed by colorectal and lung cancers. This distribution is broadly consistent with national and global cancer statistics, which identify breast, lung, colorectal, and prostate cancers as the most commonly diagnosed malignancies in Lebanon and worldwide [10,16]. According to the International Agency for Research on Cancer, breast cancer was the leading cancer among female patients in Lebanon in 2022, whereas prostate cancer ranked first among male patients, with lung and colorectal cancers also contributing substantially to the national cancer burden [10]. The relatively high proportion of patients with breast cancer observed in the present study may reflect the hospital-based recruitment strategy, the demographic composition of the study population, and the inclusion of patients receiving hospital-administered systemic therapy. Therefore, the distribution of cancer types in this study should not be interpreted as representative of the national incidence of cancer.

The present study revealed that most participants had advanced-stage disease, with 50.4% presenting with stage IV cancer and 22.0% presenting with stage III disease. This finding should be interpreted in light of the hospital-based design, which included patients receiving active systemic therapy and may therefore overrepresent individuals with advanced disease. Similar patterns of late presentation have been described in low- and middle-income countries [17]. Nevertheless, previous studies have shown that late-stage presentation is associated with greater treatment complexity, higher healthcare costs, increased hospital visits, and poorer clinical outcomes [18]. In Lebanon, these findings should be considered within the broader context of socioeconomic challenges that have been reported as potential contributors to delayed medical evaluation and late-stage presentation [9].

In the present study, more than half of the participants reported initiating treatment more than two weeks after diagnosis, and 20 patients experienced delays exceeding three months. Similar findings have been reported in Lebanon, where previous studies have documented a high proportion of advanced-stage breast cancer presentations and challenges affecting timely cancer care delivery [19]. Previous evidence has shown that prolonged delays in cancer treatment are associated with poorer survival for several cancer types [3]. However, treatment delays should be interpreted cautiously, as not all delays reflect deficiencies in care. Some may be clinically appropriate, such as those related to planned surgery, multidisciplinary treatment planning, or completion of diagnostic investigations. For example, performing surgery before systemic therapy can be a suitable, planned part of treatment, especially for early-stage or operable cancers. However, prolonged intervals between surgery and the initiation of adjuvant therapy may have clinical implications. Conversely, delays related to financing, imaging, laboratory testing, or administrative processes as observed in the current findings, may present health system challenges. In Lebanon, these challenges may be further compounded by the ongoing economic crisis and constraints on healthcare resources. The World Health Organization emphasized that timely cancer care includes not only early diagnosis but also prompt completion of diagnostic evaluation and initiation of appropriate treatment [18].

With respect to the socioeconomic profile of the study population, more than half of the participants were unemployed (57%), whereas fewer than one-third were employed. Unemployment among participants should be interpreted within the broader context of the economic and social challenges faced by patients undergoing cancer treatment, as cancer care may involve frequent healthcare visits, treatment-related symptoms, and psychological burdens that can affect employment capacity. Loss of employment or reduced work capacity may also increase vulnerability to financial hardship, particularly when treatment-related expenses are not fully covered [8].

The distribution of household income observed in this study further reflects the socioeconomic vulnerability of patients undergoing cancer treatment in Lebanon, with most participants reporting monthly household incomes between USD 200 and USD 500 or between USD 500 and USD 700. Notably, the lower household income categories reported in this study were close to the official minimum monthly wage for an individual worker in Lebanon in 2024, highlighting the limited financial resources available to some households managing the costs of cancer care [20]. These findings are important given the substantial direct and indirect costs throughout diagnosis and treatment. Previous studies have shown that cancer-related expenses may lead to out-of-pocket spending, loss of income, depletion of savings, debt accumulation, and psychological distress [8,21].

More than 80% of the participants reported a substantial decline in household income since the onset of Lebanon’s socioeconomic crisis in 2019. This finding aligns with the broader national context, where the prolonged economic crisis has been associated with reduced purchasing power and increased poverty levels [9]. For patients undergoing cancer treatment, reduced household income may represent an additional challenge given the prolonged nature of cancer care and the need for repeated healthcare encounters. The participants reported multiple coping strategies, including family support, the use of savings, asset sales, debt, and donations. The reliance on multiple mechanisms suggests that households may be combining different resources to sustain daily living and treatment-related expenses. Such strategies have been described in the literature as manifestations of financial toxicity among cancer patients and their families [6].

Findings related to health coverage further highlight gaps in financial protection among the study population. Fewer than half of the participants reported having medical insurance (43%), and insurance benefits varied, with some participants receiving coverage limited to specific components of care, such as hospitalization admission fees. Limited coverage of cancer-related services may leave patients vulnerable to substantial out-of-pocket expenses throughout the treatment pathway.

Cancer care involves multiple stages, including diagnostic evaluation, laboratory testing, genetic and molecular testing, surgery when indicated, systemic therapy, and follow-up care. Each stage may generate direct medical expenses and indirect costs for patients and their families [21]. In the present study, only 30.7% of the participants reported undergoing genetic testing. This finding should be interpreted in the context of the growing role of molecular profiling in guiding targeted therapies and immunotherapies. The European Society for Medical Oncology highlights the importance of next-generation sequencing and molecular profiling in several advanced malignancies; however, access to these technologies may vary depending on cost, availability, reimbursement mechanisms, and healthcare system capacity [22]. The proportion of patients undergoing molecular testing should therefore be interpreted in relation to cancer type, stage, and clinical indications, which were not evaluated in detail in this study. Among participants who underwent surgery, reported out-of-pocket costs varied substantially, ranging from USD 1,000 to more than USD 20,000 in some cases. Only 41.2% reported that surgical expenses were fully covered by insurance and/or the NSSF. This variability reflects differences in healthcare coverage, reimbursement eligibility, and care pathways. These findings are particularly relevant in Lebanon, where healthcare financing involves multiple public and private payers and continues to rely substantially on out-of-pocket payments [9]. Laboratory testing also represented an important component of cancer-related expenses. Although individual laboratory tests may be less costly than major interventions such as surgery or systemic therapy are, they are frequently repeated throughout the cancer care pathway for diagnosis, treatment monitoring, toxicity assessment, and follow-up. Consequently, cumulative laboratory-related expenses may contribute to the overall financial burden experienced by patients and families [10].

The most frequently reported treatment modalities in this study were chemotherapy, targeted therapy, immunotherapy, and radiotherapy, whereas hormonal therapy was not recorded among the supplied treatments. Most participants received cancer therapy as planned; however, incomplete treatment or an absence of treatment was observed among a subset of patients, particularly for chemotherapy, targeted therapy, and immunotherapy. These differences should be interpreted in light of the clinical indications, disease characteristics, and complexity of accessing newer systemic therapies. Targeted therapies and immunotherapies are often associated with higher costs and may require specific eligibility criteria and approval processes, which can influence their availability in different healthcare settings [5,22,23]. Most cancer therapies were supplied through the hospitals, while the MOPH was another important source. Some participants also reported purchasing medications directly from pharmacies. The predominance of hospitals as a treatment source should be interpreted carefully, as hospital supply does not necessarily indicate that the institution financed the medication; rather, it may reflect that treatment was administered or dispensed through the hospital. In Lebanon’s fragmented healthcare financing system, access to cancer medicines often involves multiple stakeholders, including hospitals, public institutions, insurers, and patients themselves [5]. This aligns with the Lebanese National Cancer Plan 2023-2028, which emphasizes improving access to cancer medicines and strengthening cancer care delivery [16]. Although direct pharmacy purchases represented a smaller proportion of treatment sources, they remain clinically and financially important because even limited out-of-pocket spending on cancer medications can represent a substantial burden for households [12]. Reliance on multiple financing and supply sources may support continuity of care but also reflects healthcare system fragmentation.

A high level of concordance with NCCN recommendations was observed in this study. This finding applies specifically to the selected hospital-based population included in the present study and should not be generalized to all cancer patients in Lebanon. The observed concordance suggests that treatment was largely aligned with guideline recommendations among patients who accessed specialized oncology services despite the challenging healthcare environment. However, the 15.2% rate of non-concordant care should be interpreted cautiously, as deviations from guideline recommendations do not necessarily represent suboptimal care. Treatment decisions may appropriately differ from guidelines because of clinical factors such as comorbidities, contraindications, treatment toxicity, or patient preferences. In addition, health system factors, including medication availability, reimbursement limitations, delayed approvals, or restricted access to diagnostic and molecular testing, may influence treatment choices in some settings [24]. A population-based study by Mezger et al. [25] conducted across sub-Saharan Africa reported guideline-concordant treatment in 374 patients (41.3%) among those with sufficient clinical documentation, accounting for 11.7% of the overall population-based cohort. The higher concordance observed in the present study may partly reflect differences in study design, as the current study included patients who had already accessed hospital-based oncology care and were actively receiving treatment, whereas population-based studies may capture broader barriers occurring before entry into specialized cancer services. Future research should further characterize whether non-concordant care reflects clinically justified decisions or potentially modifiable barriers within the healthcare system. Overall, these findings suggest that, among patients able to access hospital-based oncology services, guideline-concordant treatment was largely maintained despite substantial socioeconomic and healthcare system challenges. Nevertheless, the financial pathways required to obtain treatment, including reliance on multiple sources of coverage and support, highlight persistent vulnerabilities in ensuring equitable access to cancer care in Lebanon.

The current study has several notable strengths. By including five tertiary care hospitals, the study captured data from multiple clinical settings and provided a broader perspective than a single-center study. This study examined multiple dimensions of cancer care among patients actively receiving therapy.

Several limitations should be acknowledged. First, the cross-sectional study design does not allow for causal inferences or the establishment of temporal relationships between variables. Furthermore, the cross-sectional design does not allow assessment of the impact of treatment delays or financial toxicity on survival or other clinical outcomes. Additionally, the descriptive design and sample size limited the ability to perform inferential analyses and identify independent predictors of treatment delays, incomplete treatment, or financial burden. The use of convenience sampling also limits external validity, as the study population may not fully represent the broader population of cancer patients in Lebanon. Second, the hospital-based sampling strategy may have introduced selection bias. The findings may not be generalizable to all patients with cancer in Lebanon, particularly those receiving exclusively oral anticancer therapy. In addition, no participants were receiving hormonal therapy at the study sites, which may have led to an underestimation of the overall burden of treatment inaccessibility. Third, recall bias may have affected self-reported variables, including household income and treatment-related costs. However, this risk was minimized through the use of a structured questionnaire and standardized interviewer training to reduce information bias. Additionally, differences between participating hospitals in patient characteristics, treatment availability, and financing mechanisms may have influenced the findings.

The assessment of concordance with NCCN recommendations may also involve some degree of subjectivity, particularly when clinical circumstances warrant deviations from guideline-recommended treatment. Importantly, classification as non-NCCN-concordant should not necessarily be interpreted as inappropriate or substandard care, as clinically justified deviations may occur based on individual patient characteristics, treatment availability, contraindications, or other contextual factors. Finally, the relatively small sample size warrants cautious interpretation of the findings. Although considerable efforts have been made to recruit participants from multiple centers, extending the study further was not feasible because of logistical constraints and the predefined eligibility criteria, including the exclusion of non-Lebanese patients and other specified exclusion criteria. Future prospective studies should follow patients longitudinally from diagnosis through treatment initiation and evaluate treatment interruptions, quality of life, and survival outcomes to better understand the impact of financial toxicity on treatment adherence and cancer outcomes.

## Conclusions

In conclusion, guideline-concordant cancer care was largely maintained among patients who accessed hospital-based oncology services despite substantial socioeconomic and healthcare-system challenges. However, cancer care delivery occurred within a context of reduced household financial capacity, incomplete insurance coverage, treatment delays, and reliance on multiple sources of financial and treatment support. These findings highlight persistent vulnerabilities in equitable access to cancer care and the need to strengthen financial protection and care pathways within the Lebanese healthcare system. Given the hospital-based cross-sectional design, these findings should not be generalized to all cancer patients in Lebanon, and prospective studies are needed to evaluate the longitudinal impact of treatment delays and financial burden on treatment adherence and clinical outcomes.
